# Methods of Pediatric Post–COVID Condition Studies in High-Impact Journals: A Systematic Review

**DOI:** 10.1001/jamanetworkopen.2025.29659

**Published:** 2025-09-30

**Authors:** Mary Rozelle, Alyson Haslam, Vinay Prasad

**Affiliations:** 1 Department of Epidemiology and Biostatistics, University of California San Francisco, San Francisco, California; 2 Sanofi, Morristown, New Jersey; 3 now with Department of Epidemiology and Biostatistics, University of California San Francisco, San Francisco, California; 4 now with US Food and Drug Administration, Bethesda, Maryland

## Abstract

**Importance:**

Preexisting health conditions complicate post–COVID condition diagnosis in children and adolescents, while the lack of standardized clinical phenotypes challenges its definition and epidemiological estimates. Prospective studies with robust methodologies are needed to minimize bias and confounders, yet they remain scarce in high-impact factor journals.

**Objective:**

To review, characterize, and assess the methodology used in studies examining post–COVID condition in children and pediatric populations in highly cited studies, which have greater visibility and are likely to be used in informing policy.

**Evidence Review:**

Studies on PubMed and studies with high citations on Web of Science published through July 2024 were reviewed. Pediatric studies from journals with an impact factor of 5 or higher were included if they employed observational or risk-benefit designs. Studies were classified based on whether they included a SARS-CoV-2 test-negative control group or a non–test-negative group, which included studies with either no comparator or a different type of comparator. Joanna Briggs Institute and Risk of Bias in Nonrandomized Studies of Exposures tools assessed the risk of bias.

**Findings:**

Of 426 publications, 24 were analyzed; 9 studies (38%) used test-negative controls, while 15 studies (63%) did not (*P* < .001). Among studies with test-negative groups, 4 (44%) used prospective cohort designs vs 5 (33%) studies without a test-negative group. Demographic reporting of post–COVID condition cases varied. Sex was reported in 12 studies (50%), but the median (IQR) sample size was small (27 female patients [14-110]; 21 male patients [10-79]). Psychiatric history was often not reported (20 patients [83%]), as well as a lack of history of comorbidities (16 patients [67%]) or body mass index (15 patients [63%]). Among 9 test-negative control studies, 4 (44%) used matched control design, while only 1 (11%) accounted for confounders by sex-stratifying results.

**Conclusions and Relevance:**

These findings highlight the need for rigorous study designs that minimize bias and confounding, ensuring a clearer definition of pediatric post–COVID condition and its sequelae. Employing true test-negative matched controls is essential for distinguishing common symptoms and assessing risk factors, while standardizing demographic reporting strengthens comparability and ensures consistency in patient selection.

## Key Points

**Question:**

What are the characteristics of studies examining post–COVID condition in pediatric populations?

**Findings:**

In this systematic review of 24 studies, 9 (38%) used test-negative controls and 15 (63%) did not. Among test-negative control studies, 4 (44%) used prospective designs vs 5 (33%) that did not, 4 (17%) used matched controls, and 1 (11%) accounted for confounders by sex-stratification.

**Meaning:**

These findings suggest that studies examining post–COVID condition in children often lack test-negative groups and methods to minimize bias, which limits the ability to make accurate conclusions about post–COVID condition in pediatric populations.

## Teaser

This systematic review evaluates the methodology used in studies examining post–COVID condition in children and pediatric populations in highly cited studies, which have greater visibility and are likely to be used in informing policy.

## Introduction

After SARS-CoV-2 infection, children and adolescents are more likely than adults to be asymptomatic or develop mild symptoms,^1^ yet may still develop post–COVID condition. The estimated incidence of pediatric post–COVID condition varies widely, ranging from 4% to 62%, with most estimates ranging from 10% to 20%.^2^ Differences in study population selection, data collection methods, and the inclusion of test-negative control groups have resulted in variable epidemiological estimates.^2,3,4^

The growing list of post–COVID condition symptoms has made it difficult to establish a clear definition,^2^ particularly when these symptoms are not a diagnosis of exclusion. Fatigue prevalence in pediatric post–COVID condition widely varies from 3% to 87%.^5^ This underscores the need for validated health questionnaires to ensure consistent symptom reporting.^6^

The core components of the post–COVID condition definition vary and are outlined in eTable in Supplement 1. For example, the US Centers for Disease Control and Prevention (CDC) definition does not require proof of confirmed or probable SARS-CoV-2 infection.^4^ In contrast, the National Academies of Sciences, Engineering, and Medicine (NASEM) definition does.^4^ The CDC defines post–COVID condition as a broad range of symptoms that persists for at least 4 weeks after infection, although it does not specifically require symptoms to last at least 3 months, whereas NASEM formally declares post–COVID condition symptoms to include fatigue, malaise, cognitive and sleeping difficulties, and problems with taste and/or smell, bloating, and bowel movement changes with symptoms present for at least 3 months.^5^ Inconsistent terminology and definitions lead to variation in studies of post–COVID condition phenotypes.

Estimates of post–COVID condition based on electronic health records (EHR) may understate its prevalence as they capture only clinical encounters.^7^ FDA authorization of at-home diagnostic self-testing kits in November 2020^8^ further complicates data collection, especially for mildly symptomatic children and adolescents whose cases may go unrecorded in EHRs.^4,7^ Less biased estimates require testing all pediatric cases within the study population.

Given methodological variability, this systematic review examines how high-impact peer-reviewed studies define and measure pediatric post–COVID condition. Our aim was to identify pediatric post–COVID condition as a study outcome.

## Methods

### Search Strategy, Screening, and Data Source

Our study used publicly available data and did not involve individual patient data. We followed Preferred Reporting Items for Systematic Reviews and Meta-analyses (PRISMA) reporting guidelines for systematic reviews.

Two independent investigators (M.R. and A.H.) searched PubMed and Web of Science (through July 22, 2024) for studies reporting on post–COVID condition in children and adolescent populations. Our broad search strategy used keywords such as (*post-acute COVID-19 syndrome* OR *long COVID* OR *post-acute sequelae of SARS-CoV-2* OR *post-Acute COVID-19 syndrome*) AND (*children* OR *pediatric* OR *kids* OR *adolescents*) AND (*clinical study* OR *clinical trial* OR *comparative study* OR *observational study*), and (*post-acute COVID-19 syndrome* OR *long COVID* OR *post-acute sequelae of SARS-CoV-2* OR *post*-*acute COVID-19 syndrome*) AND (*child* OR *children* OR *childrens* OR *childs* OR *paediatrics* OR *pediatrics* OR *kids*) OR (*adolescences* OR *adolescency* OR *adolescent* OR *adolescent* OR *adolescence* OR *adolescents*) AND (*clinical study* OR *clinical trial* OR *comparative study* OR *observational study*). The search criteria included all articles on PubMed but were restricted to highly cited studies or hot studies (recent and highly cited studies, as defined by Web of Science) on Web of Science. Titles, abstracts, and full-text studies were independently screened by M.R. and A.H. In reviewing articles, we obtained impact factors from the journal’s website and selected articles with a 2023 impact factor of 5 or greater. We focused on these articles because they were ones more likely to be read, cited, and used to inform policy.

We included original research studies (randomized, interventional, observational, and risk-benefit) evaluating post–COVID condition symptoms in the children and adolescent population that reported post–COVID condition as an outcome. We were primarily focused on children and adolescents aged 18 years and younger, but because some organizations and countries define this population as younger than 21 years of age, we included these studies to allow for a higher number of studies. Studies could be published anytime and were not restricted to any specific geographical region.

We excluded studies not in English, duplicate records, or studies that did not address post–COVID condition as an outcome, including protocols, commentaries, reviews, qualitative studies, editorials, position articles, or meta-analyses. We excluded studies in adult populations or studies with both adult and pediatric populations, as well as those published in journals with an impact factor less than 5.0.

### Classification of Types of Comparison Groups Within Studies

We classified studies according to whether they incorporated SARS-CoV-2 test-negative controls, did not include test-negative controls, or were noncomparators. In a test-negative design approach, studies with test-negative control groups were defined as children and adolescents with a history of confirmed negative SARS-CoV-2 tests compared with children and adolescents with confirmed positive SARS-CoV-2 tests. All other studies were classified as non–test-negative groups.

The term non–test-negative groups refers to studies comparing children and adolescents with confirmed positive tests and those presumed to be test-negative, who were never tested for SARS-CoV-2 (ie, without a positive SARS-CoV-2 test), who were asymptomatic and untested, or who had previously recovered. This category also included studies that lacked a comparator group. The term healthy patients was classified as unclear due to uncertainty about their confirmed test-negative status.

### Data Extraction

From each study, descriptive variables extracted (by M.H. and A.H.) include journal name and year of publication; country; study design; dates of enrollment period; post–COVID condition framework definition; case definition present (whether COVID was tested for); method of case ascertainment; diagnostic criteria (duration of COVID symptoms and time after COVID infection); presence of sample size calculation for power analysis; assessment questionnaire to determine post–COVID condition; reported post–COVID condition symptoms; whether there was a comparator group included; whether there was matching, adjustments, or stratification of participants (by month of test, hospitalization, time period of cohort entrance, race, ethnicity, sex, age, body mass index [BMI], health insurance status, comorbidities, and psychiatric history); follow-up time; inclusion and/or exclusion criteria present; comparator class (whether they included SARS-CoV-2 test-negative controls, did not include test-negative controls, or were noncomparators); age of cases and/or comparator groups; and outcomes assessed. We also gathered baseline factors such as age and sex, BMI, comorbidities, and symptom severity.

In October 2021, the WHO established a consensus definition of post–COVID condition in adults and introduced *International Statistical Classification of Diseases and Related Health Problems, Tenth Revision* and *Eleventh Revision* codes.^9^ In contrast, definitions for children and adolescents were finalized in February 2023.^10^ Therefore, studies were classified by patient enrollment beginning before October 2021.

### Quality Assessment

To evaluate 3 areas of bias (selection, performance, and detection), we use a combination of questions from the JBI critical appraisal tool for case series studies^11^ and the Risk of Bias in Nonrandomized Studies of Exposures (ROBINS-E) assessment tool to assess the quality of studies.^12^ We adapted the reporting questions to provide a meaningful interpretation for our study. Using the JBI critical appraisal tool, we assessed whether clear inclusion and exclusion criteria were provided and whether outcomes of cases were reported through blinding to minimize selection (definitions, inclusion/exclusion criteria, and confounding) and detection biases (blinding). From the ROBINS-E tool, we examined whether confounders and unintended exposures contributed to selection and performance biases. Two independent reviewers (M.R. and A.H.) conducted assessments, with a third (V.P.) resolving discrepancies.

### Statistical Analysis

Our study’s findings are summarized using descriptive statistics in R statistical software, version 4.4.1 (R Project for Statistical Computing). We described studies using frequency and percentages for categorical variables and median with interquartile range (IQR) for numerical variables. We used χ^2^ tests and Wilcoxon-rank sum tests to determine differences for categorical and continuous variables. We used a 2-sided *P* value of .05 to determine statistical significance.

## Results

### Screening

A total of 426 titles and abstracts were screened. After excluding duplicates and non-English publications, 402 remained for full-text review. Of these, 378 did not meet the inclusion criteria, leaving 24 publications for analysis (eFigure in Supplement 1). eTable in Supplement 1 lists the definitions used in post–COVID condition studies included in this review.

### Study Characteristics

Table 1 summarizes the characteristics, symptom reporting and scoring, eligibility, post–COVID condition definitions, and test-negative control results from 24 studies.^13,14,15,16,17,18,19,20,21,22,23,24,25,26,27,28,29,30,31,32,33,34,35,36^ The study designs included cohort (12 studies^13,15,18,20,22,25,26,27,28,30,33,34^ [50%]), cross-sectional (8 studies^16,17,21,23,24,31,32,36^ [33%]), case reports, case series, and risk-benefit (4 studies^14,19,29,35^ [17%]) study design. Research originated in Europe (15 studies^15,17,18,19,21,23,24,27,28,29,31,33,34,35,36^ [63%]), North America (2 studies^20,26^ [8%]), and Asia (7 studies^13,14,16,22,25,30,32^ [29%]).

**Table 1.** General Study Characteristics

Variables, description	Studies, No. (%)		*P* value
	Studies with test-negative groups^a^ (n = 9)	Studies with non–test-negative groups^b^ (n = 15)	
Study design			
Case report or case series	0	3 (20.0)	.45
Cross-sectional	3 (33.3)	5 (33.3)	
Prospective cohort	4 (44.4)	5 (33.3)	
Retrospective cohort	2 (22.2)	1 (6.7)	
Risk-benefit analysis	0	1 (6.7)	
Continent			
Asia	3 (33.3)	4 (26.7)	.12
Europe	4 (44.4)	11 (73.3)	
North America	2 (22.2)	0	
Calculation of sample size			
No	8 (88.9)	15 (100)	.79
Yes	1 (11.1)	0	
Cases analyzed, median (IQR)	1254 (253-17 275)	62 (26-294)	.08
Age, cases in y, median (IQR)	11 (9-13)	13 (9-14)	.63
Age, comparators in y, median (IQR)	8 (6-10)	14 (10-14)	.24
Reporter of symptoms			
Carers for children and self-reported by adolescents	2 (22.2)	2 (13.3)	.95
Database or electronic health record	2 (22.2)	3 (20.0)	
Parent proxy of children	1 (11.1)	1 (6.7)	
Self-reported	2 (22.2)	4 (26.7)	
Unclear	2 (22.2)	5 (33.3)	
Reporting high-risk factors in inclusion/exclusion criteria			
No	8 (88.9)	9 (60.0)	.31
Yes	1 (11.1)	5 (33.3)	
Unclear	0	1 (6.7)	
Type of questionnaire			
Standardized and validated	5 (55.5)	7 (46.7)	.53
Nonvalidated	0	2 (13.3)	
Not specified	4 (44.4)	7 (46.7)	
Enrollment period, median (IQR), mo	6.00 (5.00-20.00)	11.00 (7.00-18.00)	.83
Enrollment after October 2021			
No	8 (88.9)	10 (66.7)	.47
Yes	1 (11.1)	5 (33.3)	
Post–COVID condition definition			
Delphi research definition	2 (22.2)	0	.37
Department of Medical Services, Ministry of Public Health, Thailand	0	1 (6.7)	
NICE	1 (11.1)	1 (6.7)	
NIH, CDC	1 (11.1)	0	
WHO	3 (33.3)	7 (46.7)	
Unclear	2 (22.2)	6 (40.0)	
Time period, median (IQR), wk	12 (7-12)	12 (8-12)	.93
Duration of symptoms, median (IQR), weeks	8 (8-8)	8 (8-8)	NA
COVID-19 determination			
Laboratory and/or clinical	0	5 (33.3)	.05
Laboratory only	9 (100)	8 (53.3)	
Not available	0	2 (13.3)	
Use of matching			
1 Criterion: age or sex	1 (11.1)	2 (13.3)	.17
2 Criteria: age and sex	0	3 (20.0)	
≥3 Criteria^c^	3 (33.3)	1 (6.7)	
Unclear	5 (55.6)	9 (60.0)	
Adjusted covariates			
1	0	0	.21
2	0	2 (13.3)	
≥3	3 (33.3)	0	
None	6 (66.7)	13 (86.7)	
Analyses stratified			
Sex	1 (11.1)	0	.32
Not indicated	0	1 (6.7)	
None	8 (88.9)	14 (93.3)	

Abbreviations: CDC, US Centers for Disease Control and Prevention; NICE, National Institute for Health and Care Excellence; NIH, National Institutes of Health; WHO, World Health Organization.

^a^ Test-negative control group: cases with confirmed negative SARS-CoV-2 tests, compared with those with confirmed positive SARS-CoV-2 cases.

^b^ Non–test-negative group: cases with confirmed positive SARS-CoV-2 tests, compared with those with presumptive SARS-CoV-2 test negative, never tested for SARS-CoV-2 (ie, without a positive SARS-CoV-2 test), asymptomatic and never tested, previously recovered individuals, or those without a comparator group.

^c^ Matching using 3 or more criteria: any combination of month of test, geography, age, sex, ethnicity, health insurance status, and psychiatry history.

Nine studies^16,20,24,25,26,27,28,32,34^ (38%) compared post–COVID condition pediatric cases with test-negative controls, and 15 studies^13,14,15,17,18,19,21,22,23,29,30,31,33,35,36^ (63%) did not (*P* < .001). Calculation of sample size for study power was present in 1 study^28^ (11%) in the test-negative control group and none among studies in the non–test-negative group. The median (IQR) sample size was 1255 (253-17 275) participants in studies with test-negative control groups vs 62 (26-294) participants in the non–test-negative group.

### Demographics

Table 2 contains demographic characteristics of post–COVID condition cases. Demographics were reported in 12 studies^20,22,23,24,26,27,28,29,33,34,35,36^ (50%), but the median (IQR) sample size was small (female patients, 27 [14-110]; male patients, 21 [10-79]). Ten studies^19,20,23,25,26,27,28,30,32,34^ (42%) did not include this information, and in 2 studies^22,29^ (8%) this was unclear.

**Table 2.** Demographics in Children and Adolescents With Post–COVID Condition (24 Studies)

Variables, description	Studies, No. (%)
Sex	
No	10 (41.7)
Yes	12 (50.0)
Unclear	2 (8.3)
No. female, median [IQR]	27.00 (13.75-109.75)
No. male, median [IQR]	20.50 (9.75-79.25)
History of psychiatric conditions	
No	20 (83.3)
Yes	2 (8.3)
Unclear	2 (8.3)
History of comorbidities	
No	16 (66.7)
Yes	7 (30.4)
Unclear	1 (4.3)
BMI^a^	
No	15 (62.5)
Yes	7 (29.2)
Unclear	2 (8.3)
No. of post–COVID condition cases by BMI, median (IQR)^a^	
<18	351 (351-351)
18.5-24.9	22 (21-1098)
≥25.0	9 (6-179)
≥30.0	5 (29-71)
≥35.0	31 (31-31)

Abbreviation: BMI, body mass index.

^a^ BMI is calculated as weight in kilograms divided by height in meters squared.

Sixteen studies^19,20,22,23,24,25,26,27,28,30,31,32,33,34,35,36^ (65%) did not report comorbidities, while 7 studies^13,14,15,16,17,18,21^ (30%) did, and 1 study^29^ (4%) was unclear. A history of having a psychiatric or mental health condition was not reported in 20 studies^13,14,15,16,18,19,20,23,24,25,26,27,28,30,31,32,33,34,35,36^ (83%), reported in 2 studies^17,21^ (8%), and unclear in 2 studies^22,29^ (8%).

Obesity, a known risk factor for post–COVID condition, was not reported in 15 studies^14,16,18,19,20,23,25,26,27,28,30,32,33,34,35^ (63%), reported in 7 studies^13,15,17,21,24,31,36^ (29%), and unclear in 2 studies^22,29^ (8%). One study^21^ enrolled underweight children and adolescents with a BMI less than 18.5.

### Differences in Reporting of Symptoms

As shown in Table 1, among 9 studies^16,20,24,25,26,27,28,32,34^ using a test-negative control design, post–COVID condition symptoms were reported by parents on behalf of their children (1 study^25^ [11%]), carers for children and self-reported by adolescents (2 studies^28,34^ [22%]), self-reported by children and/or adolescents (2 studies^16,27^ [22%]), epidemiology database or EHR (2 studies^20,22^ [22%]), or not reported or unclear (2 studies^24,26^ [22%]).

Among 15 studies^13,14,15,17,18,19,21,22,23,29,30,31,33,36^ without a test-negative control group, post–COVID condition symptoms were reported by parents on behalf of children (1 study^23^ [7%]), carers for children and/or self-reported by adolescents (2 studies^13,15^ [13%]), children and/or adolescents through self-reporting (4 studies^14,17,21,35^ [27%]), and epidemiology database or EHR (3 studies^22,29,30^ [20%]). In 5 studies^18,19,31,33,36^ (33%), the reporter of symptoms was unclear.

### Comparability of Eligibility

As shown in Table 1, 1 study^26^ with a test-negative group (11%) and 5 studies^21,22,30,35,36^ without a test-negative group (33%) included eligibility reporting on at least 1 risk factor for post–COVID condition. These high-risk factors were allergic disease, obesity, and comorbidities beyond age and sex.^37^ In contrast, 8 studies^16,20,24,25,27,28,32,34^ (89%) with test-negative controls and 9^13,14,15,17,18,19,23,29,33^ without (60%) did not report on such high-risk eligibility criteria.

### Definition of Post–COVID Condition

**Figure 1.**
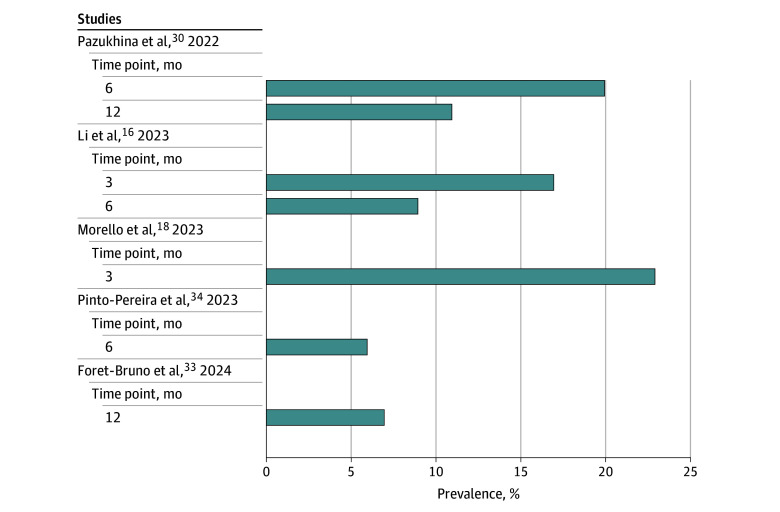
Reported Post-COVID Condition Prevalence in Children and Adolescents Among the 5 studies reporting prevalence, the mean post-COVID condition rates in children and adolescents were 20% at 3 months, 12% at 6 months, and 9% at 12 months.^15,16,17,18,19^

As shown in Table 1, 21 of 24 studies (88%) provided a definition of pediatric post–COVID condition, with 10 studies^15,17,18,21,23,24,25,27,29,30^ (42%) referencing the WHO adult definition framework, while others cited definitions from Delphi research (2 studies^16,34^ [8%]), National Institute for Health and Care Excellence (2 studies^28,36^ [8%]), National Institutes of Health/US CDC (1 study^20^ [4%]), and the diagnostic criteria from Thailand’s Department of Medical Services of the Ministry of Public Health^13^ (1 study^13^ [4%]). Additionally, this information was unclear or not reported in 8 studies^14,19,22,26,31,32,33,35^ (34%). Although most studies enrolled participants before October 2021, only 6^15,16,17,22,33,36^ of 24 studies (25%) that enrolled participants after this date applied the WHO definition. Additionally 2^13,14^ of the 24 studies (8%) used nonvalidated questionnaires to gather symptom data. While definitions of pediatric post-COVID condition varied across studies, a subset of 5 studies that reported prevalence found mean (SD) rates of 20% (4.38%) at 3 months, 12% (9.72%) at 6 months, and 9% (2.83%) at 12 months (Figure 1).

Among the 9 studies using test-negative control group,^16,20,24,25,26,27,28,32,34^ the median (IQR) duration from infection or positive COVID-19 test to persistent symptoms, based on the selected post–COVID condition definition framework, was 12 (7-12) weeks, with symptoms lasting a median (IQR) duration of 8 (8-8) weeks. Among the 24 studies, COVID-19 status was determined through laboratory testing 17 studies^13,16,18,19,20,21,23,24,25,26,27,28,30,32,33,34,35^ (71%), while 5 studies^14,15,17,22,36^ (21%) required both laboratory and clinical criteria. Two studies^29,31^ (8%) did not report how COVID-19 status was ascertained.

### Similarity of Symptoms for Post–COVID Condition

Prespecified symptoms were collected using standardized and validated questionnaires in 11^15,16,17,21,23,25,27,28,30,33,34^ of 24 studies (46%) as shown in Table 1. These questionnaires include Chalder Fatigue, Patient-Reported Outcomes Measurement Information System Paediatric Fatigue Scale, Children’s Somatic Symptoms Inventory, International Severe Acute Respiratory and Emerging Infection Consortium, Pediatric Quality of Life Inventory, and the COVID-19 Health and Well-being Follow Up Survey for Children. Two^13,14^ of 24 studies (8%) used nonvalidated questionnaires, 1 developed by members of the Royal College of Pediatricians of Thailand,^13^ and another relied on freely reported symptoms data later organized into a survey tool by the team.^14^ Data collection methods were not specified in 11 studies^18,19,20,22,24,26,29,31,32,35,36^ (46%).

### Confounder Accounting Through Matching, Covariate Adjustment, or Stratification

Among the 9 studies with test-negative control groups listed in Table 1, 4^25,28,32,34^ (44%) matched on factors such as month of testing, geography, age, sex, ethnicity, health insurance, and psychiatry history. A total of 4^24,27,28,34^ (44%) were conducted in Europe. In comparison, among studies without test-negative groups, 5^13,15,18,30,33^ (33%) used a prospective design, while the majority of studies (11 studies^15,17,19,21,23,28,29,31,33,35,36^ [73%]) were based in Europe. Matching procedures were unclear in 5 studies^16,20,24,26,27^ (56%). Three studies^20,26,32^ (22%) performed covariate adjustments using 3 or more variables, including combinations of age, sex, race, ethnicity, geography, BMI, and comorbidities. The remaining 6 studies^16,24,25,27,28,34^ (67%) did not report on any covariate adjustments. Furthermore, 1 study^32^ (11%) stratified results based on sex.

###  Possibility That Participants in Studies With Test-Negative Control Groups Could Have Been Test-Positive 3 Months Prior

Among test-negative control groups, 7^16,24,25,27,28,32,34^ (78%) had a documented history of never testing positive for SARS-CoV-2. Only 1 study^27^ (11%) reported excluding those with test positivity more than 28 days prior. The majority of studies with test-negative groups did not provide further details about the timing of prior testing (8 studies^16,20,24,25,26,28,32,34^ [89%]).

### Overall Study Quality

A risk of bias assessment was performed (Table 3). To evaluate selection bias, we examined whether inclusion and exclusion criteria specified at least 1 risk factor for post–COVID condition in children and adolescents, such as a history of allergic conditions, obesity, or other comorbidities.^37^ Six^21,22,26,30,35,36^ of the 24 studies (25%) included these risk factors as part of their inclusion criteria, whereas 17 studies^13,14,15,16,17,18,19,20,23,24,25,27,28,29,32,33,34^ (71%) did not. One study^31^ (4%) lacked sufficient detail for evaluation. When assessing exclusion criteria beyond age and COVID status, 12 studies^20,22,23,24,26,27,28,29,33,34,35,36^ (50%) reported such criteria, 11^13,14,15,16,17,18,19,21,25,30,32^ (46%) did not, and 1^31^ (4%) was unclear.

**Table 3.** Risk of Bias Assessments for Observational Studies (24 Studies)

Bias types, description	Studies, No. (%)
Selection bias	
Risk factor defined in inclusion/exclusion criteria	
No	17 (70.8)
Yes	6 (25.0)
Unclear	1 (4.2)
Comorbidities included in the post–COVID condition definition	
Comorbidities	1 (4.2)
Asthma	1 (4.2)
Diabetes, chronic lower respiratory disease, chronic kidney disease, nicotine dependence, human immunodeficiency virus disease, cerebrovascular diseases, hypertension, sickle-cell disorders, mental diseases, neoplasms, liver disease, overweight and obesity, transplanted organ and tissue status	1 (4.2)
Neurological comorbidities, allergic respiratory disease, obesity	1 (4.2)
Obstructive sleep apnea, obesity, hypertension, diabetes, asthma	1 (4.2)
Preexisting cardiovascular, pulmonary, endocrinological, rheumatological, or oncological diseases	1 (4.2)
None	17 (70.8)
Unclear	1 (4.2)
Exclusion criteria present	
No	11 (45.8)
Yes	12 (50.0)
Unclear	1 (4.2)
Minimize confounders	
No	12 (50.0)
Yes	12 (50.0)
Performance bias	
Consider unintended exposure	
No	1 (4.2)
Yes	23 (95.8)
Detection bias	
Blinding investigators	
No	24 (100)

To minimize performance bias in these studies, study investigators expressed unintended exposures that may have biased results in 23 studies^13,14,15,16,17,18,20,21,22,23,24,25,26,27,28,29,30,31,32,33,34,35,36^ (96%). Lastly, to prevent detection bias and maintain internal validity, none of the studies incorporated investigator blinding or outcome assessor masking between groups.

## Discussion

Our systematic review identified that most pediatric post–COVID condition studies lacked test-negative control groups, limiting clarity on symptom specificity and risk factors. Incorporating study designs with true test-negative controls strengthens the ability to define post–COVID condition sequelae in the pediatric population. Our recommendation will promote more accurate diagnosis and informed clinical care globally.

However, even among studies that did include test-negative groups, limitations in verifying prior infection status may have introduced bias. It is possible that studies using test-negative groups may have inadvertently included individuals who had previously tested positive for SARS-CoV-2 and who may have been predisposed to developing post–COVID condition. In our study, test history was not available in 2 studies^20,26^ (22.2%) with test-negative controls. The gap in test history reporting raises concerns about the reliability of test-negative classifications, especially in estimating post–COVID condition prevalence and outcomes in children and adolescents.

Our study highlighted key methodological differences in study design, sample size justification, and geographic distribution across studies. One study^28^ (11%) in the test-negative control group conducted a sample size calculation for power analysis, whereas none did so in the non–test-negative group. Of the studies with test-negative control groups, 4^25,27,28,34^ (44%) employed prospective cohort designs, and 4^24,27,28,34^ (44%) were conducted in Europe. In comparison, among studies without test-negative groups, 5^13,15,18,30,33^ (33%) used a prospective design, while the majority of studies (11 studies^15,17,19,21,23,28,29,31,33,35,36^ [73%]) were based in Europe. The limited representation across continents may warrant caution in interpreting prevalence as the trajectory of the SARS-CoV-2 virus outbreak and management could impact findings.^38,39^

We found heterogeneity in data collection tools used to define post–COVID condition symptoms. Although most studies enrolled participants before October 2021, only 6 of 24 studies^15,16,17,22,33,36^ (25%) that enrolled participants after this date applied the WHO definition.^9^ Additionally, 2 of the 24 studies^13,14^ (8%) used nonvalidated questionnaires to gather symptom data. These inconsistencies underscore the urgent need for standardized, validated tools and reliable pediatric post–COVID condition definitions.

Finally, we identified demographic data of pediatric cases with post–COVID condition. Although sex was reported in most post–COVID condition cases (50%), the median sample size of participants in these studies was small. Although 20 studies^13,14,15,16,18,19,20,23,24,25,26,27,28,30,31,32,33,34,35,36^ (83%) did not report psychiatric history of cases, 16^19,20,23,24,25,26,27,28,30,31,32,33,34,35,36^ (67%) failed to report comorbidities, and 15^14,16,18,19,20,23,25,26,27,28,30,32,33,34,35^ (63%) did not provide information on BMI. Ideally, selecting cases with similar risk profiles as test-negative matched controls would be prudent for assessing the potential development of post–COVID condition symptoms. Our findings emphasize the importance of clearly defining the demographics of these cases so comparability of patient selection would be possible.

Among the 9 studies with test-negative groups, 4^25,28,32,34^ performed matching and 3^20,26,32^ (33%) adjusted for covariates, helping to mitigate confounding. Only 1 study^32^ (11%) reduced confounding in the analysis by sex-stratifying results. Overall, efforts to address confounding were limited.

Most inclusion and exclusion criteria (17 studies^13,14,15,16,17,18,19,20,23,24,25,27,28,29,32,33,34^ [71%]) did not account for at least 1 risk factor for post–COVID condition in children and adolescents, such as a history of allergic conditions, obesity, or other comorbidities.^37^ While many studies (12 studies^20,22,23,24,26,27,28,29,33,34,35,36^ [50%]) included exclusion criteria, 11^13,14,15,16,17,18,19,21,25,30,32^ (46%) did not. As a result, it is challenging to determine whether baseline differences would further worsen confounding or selection bias.

We assessed whether investigators were blinded, particularly those involved in the assessment or analysis. None of the studies implemented blinding, which may introduce detection bias.

### Strengths and Limitations

A key strength is our systematic evaluation of the methods to assess the rigor of existing studies and standards shaping scientific understanding and public perceptions of pediatric post–COVID condition. There are, however, some limitations. We selected articles from high-impact journals, which likely feature higher-quality studies. Additionally, we relied on the information provided by the authors. Due to word limits and reporting expectations, some relevant details may not have been included. Moreover, our results may be interpreted cautiously due to the small sample size of high-impact factor journals.

## Conclusions

In this systematic review, most studies did not use formal test-negative controls, suggesting that post–COVID condition symptoms may be nonspecific and/or not directly attributable to prior infection with the SARS-CoV-2 virus. Standardizing demographic reporting strengthens comparability and ensures patient selection consistency.

## Notes

Supplement 1. **eFigure.** PRISMA Diagram With Exclusion Criteria Used for the Screening Process **eTable.** Definitions Used in High-Impact Studies Assessing Post–COVID Condition in Children and Adolescents

Supplement 2.
Data Sharing Statement
