# Supplementary material for: Methods of Pediatric Post–COVID Condition Studies in High-Impact Journals: A Systematic Review
Source: JAMA Netw Open. 2025 Sep 30;8(9):e2529659. doi: 10.1001/jamanetworkopen.2025.29659 (PMC12485639; doi:10.1001/jamanetworkopen.2025.29659)
Supplement: Supplement 1. — eFigure. PRISMA Diagram With Exclusion Criteria Used for the Screening Process eTable. Definitions Used in High-Impact Studies Assessing Post–COVID Condition in Children and Adolescents [file jamanetwopen-e2529659-s001.pdf]

Supplemental Online Content

Rozelle M, Haslam A, Prasad V. Methods of pediatric long COVID studies in high-impact journals: a systematic review. *JAMA Netw Open*. 2025;8(8):e2529659. doi:10.1001/jamanetworkopen.2025.29659

eFigure. PRISMA Diagram With Exclusion Criteria Used for the Screening Process
eTable. Definitions Used in High-Impact Studies Assessing Post-COVID Condition in Children and Adolescents

This supplemental material has been provided by the authors to give readers additional information about their work.

© 2025 Rozelle M et al. *JAMA Network Open*.

eFigure. PRISMA Diagram With Exclusion Criteria Used for the Screening Process

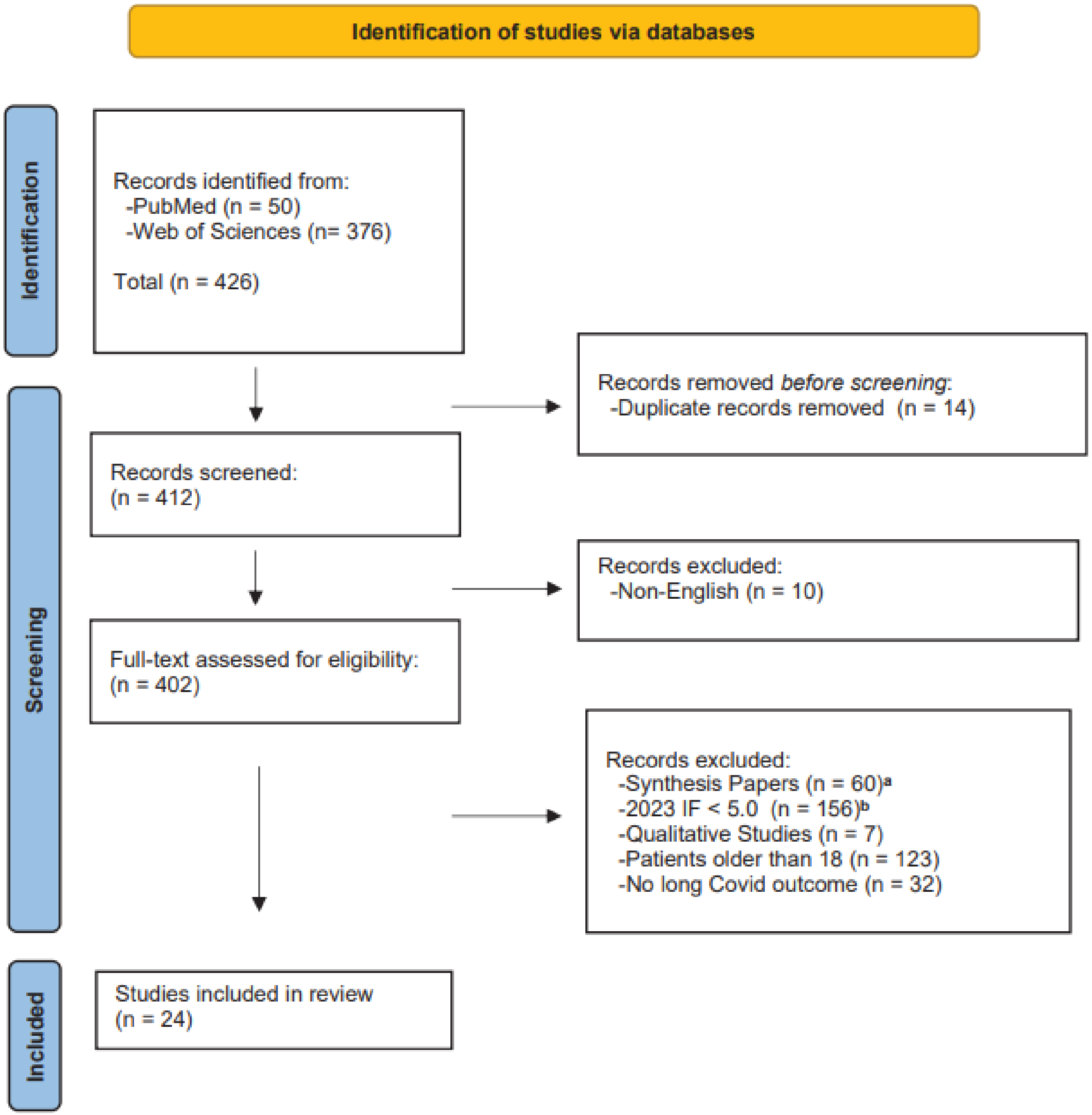

Note: Out of 426 identified studies, 24 were included in this analysis after applying the inclusion and exclusion criteria.

a. Synthesis Papers include reviews (n= 37), commentary (n= 1), protocols (n= 17), policies (n= 4), position paper (n= 1)
b. 2023 Impact Factor rating (Clarivate) < 5.0 reflects journal's citation

© 2025 Rozelle M et al. *JAMA Network Open*.

eTable. Definitions Used in High-Impact Studies Assessing Post-COVID Condition in Children and Adolescents

**Framework Definition of Long COVID**	**Description**
Delphi research definition[a]	"Include at least 1 physical symptom persisting for a minimum of 12 weeks after the initial confirmed infection that may continue or develop after infection, cannot be explained by an alternative diagnosis, has an effect on everyday functioning, and may fluctuate or relapse over time."
Diagnostic criteria, Dept of Medical Sciences Ministry of Public Health, Thailand[b]	Unclear
NICE[c]	"Signs and symptoms that develop during or after an infection consistent with COVID-19 continue for more than 12 weeks and are not explained by an alternative diagnosis. It usually presents with clusters of symptoms, often overlapping, which can fluctuate and change over time and affect any system in the body. Post-COVID-19 syndrome might be considered before 12 weeks while the possibility of an alternative underlying disease is also being assessed."
US NIH[d]	"Post-acute infected children and young adults who reported having a COVID infection more than 30 days before enrollment" were categorized as having a high, medium, or low probability of PASC."
US CDC[e]	"An umbrella term for the wide range of physical and mental health consequences experienced by some patients that are present 4 or more weeks after SARS-CoV-2 infection, including patients who had initial mild or asymptomatic acute infection."
NASEM[e]	"Occurs after SARS-CoV-2 infection and is present for at least 3 months as continuous, relapsing and remitting, or progressive disease state that affects one or more organ systems."
WHO, Adult[f]	"Continuation or development of new symptoms 3 months after the initial infection, with symptoms lasting for at least 2 months with no other explanation."
WHO, Children and Adolescents[g]	"It occurs in individuals with a history of confirmed or probable SARS-CoV-2 infection when symptoms last at least 2 months, initially occurring within 3 months of acute COVID-19. Current evidence suggests that symptoms more frequently reported in children and adolescents with post-COVID-19 conditions compared with controls are fatigue, altered smell (anosmia), and anxiety. Other symptoms have also

© 2025 Rozelle M et al. *JAMA Network Open*.

	been reported. Symptoms generally impact everyday functioning, such as changes in eating habits, physical activity, behavior, academic performance, social functions (interactions with friends, peers, and family), and developmental milestones. Symptoms may be new onset following initial recovery from an acute COVID-19 episode or persist from the initial illness. They may also fluctuate or relapse over time. Workup may reveal additional diagnoses, but this does not exclude the diagnosis of post-COVID-19.”

Note: Abbreviation (NASEM, National Academies of Sciences, Engineering, and Medicine)

a. Stephenson T, Allin B, Nugawela MD, et al. Long COVID (post-COVID-19 condition) in children: a modified Delphi process. *Arch Dis Child*. 2022;107(7):674-680. doi:10.1136/archdischild-2021-323624

b. Jarupan M, Jantarabenjakul W, Jaruampornpan P, et al. Long COVID and Hybrid Immunity among Children and Adolescents Post-Delta Variant Infection in Thailand. *Vaccines (Basel)*. 2023;11(5):884. Published 2023 Apr 23. doi:10.3390/vaccines11050884

c. National Institute for Health and Care Excellence (NICE). COVID-19 Rapid Guideline: Managing the Long-term Effects of COVID-19. Published 18 December 2020. https://www.nice.org.uk/guidance/ng188/chapter/1-Identification

d. Gross R, Thaweethai T, Rosenzweig EB, et al. Researching COVID to enhance recovery (RECOVER) pediatric study protocol: Rationale, objectives and design. Preprint. *medRxiv*. 2023;2023.04.27.23289228. Published 2023 May 12. doi:10.1101/2023.04.27.23289228

e. Ely EW, Brown LM, Fineberg HV; National Academies of Sciences, Engineering, and Medicine Committee on Examining the Working Definition for Long Covid. Long Covid Defined. *N Engl J Med*. 2024;391(18):1746-1753. doi:10.1056/NEJMsb2408466

f. World Health Organization. Post COVID-19 Condition (Long COVID). Published 6 October 2021. https://www.who.int/europe/news-room/fact-sheets/item/post-covid-19-condition

g. World Health Organization. A Clinical Case Definition for Post COVID-19 Condiiton in Children and Adolescents By Expert Consensus. Published 16, February 2023. https://iris.who.int/bitstream/handle/10665/366126/WHO-2019-nCoV-Post-COVID-19-condition-CA-Clinical-case-definition-2023.1-eng.pdf?sequence=1

© 2025 Rozelle M et al. *JAMA Network Open*.
