# Supplementary material for: Methods of Pediatric Post–COVID Condition Studies in High-Impact Journals: A Systematic Review
Source: JAMA Netw Open. 2025 Sep 30;8(9):e2529659. doi: 10.1001/jamanetworkopen.2025.29659 (PMC12485639; doi:10.1001/jamanetworkopen.2025.29659)
Supplement: Supplement 2. — Data Sharing Statement [file jamanetwopen-e2529659-s002.pdf]

Data Sharing Statement

Rozelle. Methods of Pediatric Post-COVID Condition Studies in High-Impact Journals. *JAMA Netw Open*. Published August 29, 2025. doi:10.1001/jamanetworkopen.2025.29659

Data
Data available: No
